# Regulatory T cells and peripheral immune tolerance: A perspective on the 2025 Nobel Prize

**DOI:** 10.1515/jtim-2026-0053

**Published:** 2026-06-13

**Authors:** Pu Liang, Xuefeng Huang, Jianmei W. Leavenworth, Xi Wang

**Affiliations:** National Key Laboratory of Intelligent Tracking and Forecasting for Infectious Diseases, Beijing Ditan Hospital, Capital Medical University, Beijing, China; Beijing Institute of Infectious Diseases, Beijing, China; Beijing Key Laboratory of Viral Infectious Diseases, Beijing Ditan Hospital, Capital Medical University, Beijing, China; National Center for Infectious Diseases, Beijing Ditan Hospital, Capital Medical University, Beijing, China; Department of Neurosurgery, University of Alabama at Birmingham, Birmingham, AL, USA; The O’Neal Comprehensive Cancer Center, University of Alabama at Birmingham, Birmingham, AL, USA; Department of Microbiology, University of Alabama at Birmingham, Birmingham, AL, USA; Department of Oncology, Capital Medical University, Beijing, China

## Introduction: Laureates and key discoveries

The 2025 Nobel Prize in Physiology or Medicine has been jointly awarded to Dr. Mary E. Brunkow and Dr. Fred Ramsdell of the United States, and Dr. Shimon Sakaguchi of Japan, for their seminal discoveries elucidating the mechanism of peripheral immune tolerance.^[1]^ Through complementary research, the laureates have collectively unraveled a fundamental immunological mystery: how the immune system distinguishes self from non-self, effectively eliminating pathogens while preventing autoimmunity. Their pivotal work identified regulatory T cells (Tregs), a specialized subset of immune cells that serve as critical sentinels, constantly shaping and restraining immune responses to preserve self-tolerance and prevent autoimmunity. The Nobel Assembly underscored the transformative impact of this discovery, stating that it “fundamentally transformed our understanding of immune system regulation, explaining why most individuals do not suffer from severe autoimmune diseases”.

## The conceptual evolution of immunological tolerance

Before these Nobel laureates’ seminal work, the prevailing view focused on central immune tolerance—a “self-check” within the thymus designed to eliminate self-recognizing T cells during development. However, this model proved incomplete, as some potentially autoreactive T cells could still escape thymic censorship and migrate to peripheral tissues without provoking widespread autoimmunity. This paradox highlighted a critical question: what mechanisms restrained these “escaped” self-recognizing lymphocytes in the periphery. Although the existence of “suppressor T cells” had been hypothesized, the idea was marginalized due to insufficient mechanistic evidence and technical limitations, causing the field to stagnate for years.

## Pivotal discoveries by the laureates

Through complementary and ingenious investigations, Sakaguchi, Brunkow, and Ramsdell revolutionized the landscape of immunological tolerance. Their pivotal contributions are summarized in the Table 1.

**Table 1:** Key discoveries and their scientific impact

Investigator	Key finding	Year	Scientific significance
Shimon Sakaguchi	Identification and characterization of Tregs^[2]^	1995	Provided the first direct evidence for the existence of peripheral immune tolerance
Mary Brunkow & Fred Ramsdell	Discovery that FOXP3 gene mutations cause autoimmune pathology^[3]^	2001	Established the genetic foundation for Treg-mediated immunosuppressive function
Shimon Sakaguchi	Demonstration of FOXP3 as the master genetic regulator of Treg development^[4]^	2003	Unified the cellular discovery with its underlying molecular mechanism, cementing the Treg paradigm

Tregs: regulatory T cells; FOXP3: forkhead box P3

Sakaguchi was inspired by a paradoxical observation: neonatally thymectomized mice developed severe autoimmune diseases rather than immunodeficiency. Subsequent experiments revealed that transferring mature T cells from healthy mice prevented autoimmunity in thymectomized recipients, suggesting that certain mature T cells possess immunoregulatory functions. Following a decade of research, Sakaguchi identified a novel T cell population expressing CD4 and CD25 in 1995, which he named “regulatory T cells (Tregs)”.^[2]^

Simultaneously, Brunkow and Ramsdell investigated the “scurfy” mouse model, which displayed severe, lethal autoimmune disease and survived only a few weeks. The exclusive incidence in male mice suggested an X-linked genetic basis.^[5]^ Through rigorous genetic mapping, they identified in 2001 the causative mutation in the X-chromosomal gene *Foxp3*, which encodes a transcription factor. They further demonstrated that mutations in the human homologous gene cause a similar rare and fatal inherited autoimmune disorder termed the immune dysregulation, polyendocrinopathy, enteropathy, X-linked (IPEX) syndrome.^[3]^

In 2003, Sakaguchi’s team unified these discoveries by proving that FOXP3 is specifically expressed in Tregs and is essential for their development. This work established the molecular basis of Treg biology, ushering in a new era of systematic functional and mechanistic exploration in this field.^[4]^

## Reconceptualizing immunological theory

These Nobel laureates’ discoveries have fundamentally reshaped our understanding of immune system regulation. They established that maintaining immune homeostasis requires not only central tolerance mechanisms in the thymus but also synergistic collaboration with peripheral immune tolerance. The peripheral immune tolerance is mediated by Tregs, which proactively suppress the activation of autoreactive T cells, providing a critical dual safeguard against autoimmunity. Importantly, central tolerance is not absolute, eliminating only approximately 60%-70% of self-recognizing T cells during thymic development.^[6]^ The remaining self-reactive cells are controlled by Treg-mediated peripheral tolerance, preventing immune attack against nearly one-third of peripheral self-antigens. This highlights the indispensable role of peripheral tolerance in preserving immune equilibrium.

## Expanding clinical horizions

The discovery of Tregs has revolutionized therapeutic strategies for multiple diseases, prompting numerous clinical trials worldwide. In autoimmune diseases like type 1 diabetes, rheumatoid arthritis, and lupus,^[7,8]^ strategies such as low-dose interleukin-2 (IL-2) to promote Treg expansion, or adoptive Treg transfer, have shown promising efficacy in re-establishing immune homeostasis. In transplantation, Tregs offer the potential to suppress graft rejection, with chimeric antigen receptor (CAR)-Treg technology showing promise for achieving antigen-specific tolerance.^[9]^ In cancer immunotherapy, anti-cytotoxic T lymphocyte-associated antigen-4 (anti-CTLA-4) antibodies primarily deplete intratumoral Tregs *via* Fc gamma receptor (FcγR)-dependent mechanisms, rather than through checkpoint blockade alone. Given that systemic Treg depletion may trigger autoimmunity, developing localized and selective Treg suppression strategies is of critical clinical importance.^[10]^ Collectively, these strategies underscore the potential of immunomodulatory precision medicine.

## Future directions and implications

Research on regulatory T cells still faces significant challenges. These include elucidating their functional heterogeneity, deciphering tissue-specific regulatory mechanisms, and determining how to precisely modulate Treg function in autoimmune diseases and cancer immunotherapy. Furthermore, the long-term safety and efficacy of Treg-based cellular therapies require extensive clinical validation. Key parameters, such as cell source, expansion protocols, timing of reinfusion, and *in vivo* persistence, demand rigorous standardization and optimization. Emerging biological technologies are thus providing powerful tools to address these complex challenges. Single-cell multi-omics enables in-depth resolution of Treg functional heterogeneity; clustered regularly interspaced short palindromic repeats (CRISPR)-based editing and epigenetic modulation allow precise tuning of stability and suppressive programs, while organoid systems and humanized models provide physiologically relevant platforms to test tissue-specific mechanisms and durability. The integrated application of these technologies is expected to drive breakthroughs in key bottlenecks in Treg research.

These advances in understanding Treg biology, coupled with the Nobel Committee’s earlier recognition of microRNA-mediated regulation in 2024, underscore a significant shift in the life sciences: from primarily descriptive studies to a systematic exploration of regulatory networks. The laureates’ work underscores that groundbreaking discoveries often arise from probing anomalous phenomena, evolving through rigorous observation, mechanistic understanding, and eventual acceptance, even in the face of initial skepticism. These achievements demonstrate that paradigm-shifting discoveries rely on prolonged data accumulation and rigorous, multidimensional validation to establish scientific consensus. With continued advances in technology and mechanistic understanding, novel immunomodulatory strategies are poised to profoundly transform the treatment landscape for autoimmune disorders, cancer, and transplantation medicine in the coming years.

Looking ahead, the Nobel Prize in Physiology or Medicine increasingly highlights an integrated continuum of innovation, from mechanistic discovery to clinical translation. Building on recent Nobel Prize-recognized achievements in gene editing, immune modulation, and mRNA technology, future transformative discoveries are likely to emerge. These may include curative therapies for cystic fibrosis, a deeper understanding of the innate immune cyclic guanosine monophosphate-adenosine monophosphate (GMP-AMP) synthase-stimulator of interferon genes (cGAS-STING) pathway, and breakthroughs in non-invasive prenatal testing, representing landmark progress in targeted therapy, fundamental immunology, and diagnostic innovation. These fields are deepening our understanding of life’s fundamental mechanisms. Ultimately, these breakthroughs, together with the laureates’ discoveries, exemplify the core mission of advancing science for the benefit of humanity, paving the way for transformative interventions against major diseases.
